# Trends in the Prevalence and Development of Alzheimer’s Disease Among the Elderly Chinese Population: A Systematic Review

**DOI:** 10.31083/RN36394

**Published:** 2025-07-28

**Authors:** Gui-Zhi Han, Zhao-Hui Liang, Li-Yan Guo, Miao-Miao Liu

**Affiliations:** ^1^School of Public Health, Jining Medical University, 272013 Jining, Shandong, China

**Keywords:** Alzheimer disease, prevalence, systematic review, meta-analysis, China, enfermedad de Alzheimer, prevalencia, revisión sistemática, metaanálisis, China

## Abstract

**Introduction:**

Dementia is a major global health challenge, with Alzheimer’s disease (AD) being the most common cause.In China, due to its large and aging population, AD poses a significant threat. Although systematic reviews on the prevalence of dementia in the Chinese population exist, relatively few have specifically targeted AD. This study aimed to analyze the prevalence of AD among the population aged 60 years and older in China from 2014 to 2024.

**Methods:**

A literature search on the prevalence of AD in China was conducted. A meta-analysis was performed using Stata version 16.0. The *I*^2^ was used to assess heterogeneity. The random-effects model was used to calculate the pooled effect size. Subgroup analyses were conducted based on different characteristics. Meta-regression was used to explore the sources of heterogeneity and identify the factors that significantly affect the effect size. Funnel plots and Egger’s test were utilized to evaluate publication bias.

**Results:**

A total of 23 studies were included, with a total sample size of 307,415, including 13,662 patients with AD. The results of the meta-analysis showed that the prevalence of AD among the elderly Chinese population was 5.4% (95% CI: 4.7%–6.2%). The results of the meta-regression indicated that factors such as female sex, advanced age, low educational level, rural residence, and geographical region are the main factors influencing the prevalence of AD.

**Conclusion:**

In the past decade, the prevalence of AD among people over 60 years of age in China was approximately 5.4%, which is a major public health problem for China.

## Abstract (es)

**Introducción:**

La demencia es un problema de salud importante a nivel mundial, y el tipo más común es la enfermedad de Alzheimer (EA). En China, debido a su gran población y al envejecimiento de la misma, la EA supone una amenaza de gran envergadura. Aunque existen revisiones sistemáticas sobre la prevalencia de la demencia en la población china, son relativamente pocas las que se centran específicamente en la EA. El objetivo de este estudio fue analizar la prevalencia de la EA entre la población de 60 años o más en China entre los años 2014 y 2024.

**Métodos:**

Se realizó una búsqueda bibliográfica sobre la prevalencia de la EA en China. El metaanálisis se llevó a cabo utilizando Stata, versión 16.0. Se empleó el I² para evaluar la heterogeneidad. Se utilizó el modelo de efectos aleatorios para calcular el tamaño del efecto combinado. Se realizaron análisis de subgrupos de acuerdo con diferentes características. Se empleó la metarregresión para explorar las fuentes de heterogeneidad e identificar los factores que afectan significativamente al tamaño del efecto. Se emplearon gráficos de embudo y la prueba de Egger para evaluar el sesgo de publicación.

**Resultados:**

Se incluyeron un total de 23 registros, con un tamaño de muestra total de 307.415, incluidos 13.662 pacientes con enfermedad de Alzheimer. Los resultados del metaanálisis indicaron que la prevalencia de la enfermedad de Alzheimer entre la población anciana china era del 5,4 % (IC del 95 %: 4,7 %–6,2 %). Los resultados de la metarregresión indicaron que factores como el sexo femenino, la edad avanzada, el bajo nivel educativo, la residencia rural y la región geográfica son los principales factores que influyen en la prevalencia de la EA.

**Conclusión:**

En la última década, la prevalencia de la enfermedad de Alzheimer entre las personas mayores de 60 años en China fue de aproximadamente el 5,4 %, un hecho que se ha convertido en un importante problema de salud pública en este país.

## 1. Background

Alzheimer disease (AD) is a progressive neurodegenerative disorder characterized by gradual loss of memory, cognitive abilities, and behavioral functions. It is the most common form of dementia, affecting millions of people worldwide and causing significant impairment in daily life activities [1, 2]. AD is a devastating condition that significantly affects the social, occupational, and daily lives of elderly individuals, imposing a heavy burden on their families and on society. With the increasing aging population worldwide, it has become a major public health concern. The China Alzheimer Disease Report 2021 showed that the standardized prevalence of AD and other dementias in China was 788.3 per 100,000 in 2019, which was higher than the global standardized prevalence of AD and other dementias of 682.5 per 100,000 [3]. In recent years, the process of global aging has accelerated, and the aging of China’s population has reached a relatively high rate, showing a trend of accelerated development. With an increase in aging, the number of elderly people with cognitive impairment is also increasing rapidly. Research indicates that by the middle of the 21st century, the elderly population in China will increase to 400 million, and the number of patients with dementia will reach 20 million [4]. Numerous systematic reviews have analyzed the prevalence of dementia among the elderly population in China. However, current information regarding the prevalence of AD is mostly fragmented and regional.

Therefore, this study systematically reviewed the studies on the prevalence of AD among the elderly in China published in the past decade, to analyze the development trend of AD prevalence, to provide data support for formulating public health service policies for the elderly, and carrying out public health service work in the future.

## 2. Methods

### 2.1 Information Source and Search Strategy

This systematic review was conducted in accordance with the Preferred Reporting Items for Systematic reviews and Meta-Analyses (PRISMA) 2020 guidelines [5]. The PRISMA checklist is available in the **Supplementary materials**. The protocol of this review had been registered in PROSPERO (CRD42024629531). From January to June 2024, a systematic search was conducted on the literature regarding the prevalence of AD in the elderly in China, published from January 1, 2014, to June 30, 2024, in CNKI, Wan Fang Database, VIP Database, PubMed, and Web of Science. The search terms included “Alzheimer Disease”, “Dementia”, “epidemiology study”, “prevalence”, “China”, and “Chinese”. Additionally, literature tracing was used to further search for relevant data.

### 2.2 Literature Screening Criteria

***Inclusion criteria:*** (1) The subjects in the studies are elderly individuals aged ≥60 years in China; (2) There is a clear sample size (sample size ≥2000), the number of patients or prevalence; (3) The publication date is from January 2014 to June 2024; (4) Original cross-sectional survey; (5) the diagnostic criteria and basis of AD are described in the article.

***Exclusion criteria:*** (1) Duplicate publication or incomplete information; (2) non-Chinese region studies; (3) Published before 2014; (4) The research objects were non-community elderly, veteran cadres, and elderly people in welfare institutions; (5) Review or non-cross-sectional survey.

### 2.3 Information Extraction Process

Two researchers independently screened the articles by reading the titles and abstracts and then conducted a secondary screening by reading the full texts. Unqualified studies were excluded based on inclusion and exclusion criteria. In case of controversial studies, the research team discussed and decided whether to include them. Microsoft Excel 2016 (version 16.0, Microsoft Corporation, Redmond, WA, USA) was used to establish an information extraction database, and the following contents were extracted from the literature: first author, publication year, study region, diagnostic criteria, subject age, sex, urban-rural status, education level, sampling method, sample size, number of cases, and prevalence of AD.

### 2.4 Literature Quality Evaluation

The Agency for Healthcare Research and Quality (AHRQ) scale was used to evaluate the quality of the included studies (11 items) [6, 7]. Each item was scored as 0 points for ‘NO’ or ‘UNCLEAR’ and 1 point for ‘YES’. A total score of ≤3 is classified as low quality, 4–7 is medium quality, and ≥8 is high quality.

### 2.5 Statistical Analysis

Meta-analysis was performed using Stata software (version 16.0, Stata Corp LLC, College Station, TX, USA). Heterogeneity was evaluated using an inconsistency index (*I*^2^). In cases where *I*^2^ ≥50%, indicating substantial heterogeneity, the random-effects model would be selected to calculate the pooled prevalence and 95% confidence intervals (95% CI) of AD [8]. Subgroup analysis was conducted to explore the sources of heterogeneity and evaluate the influence of different demographic characteristics on the effect size. Meta-regression was employed to identify the sources of heterogeneity, and clarify the factors that significantly affect the effect size and the extent of their influence. Publication bias was evaluated using the Egger’s test and a funnel plot. The trim-and-fill method was employed to eliminate publication bias. A sensitivity analysis was conducted by excluding each study individually. Statistical significance was set at *p* < 0.05 (two-tailed test).

## 3. Results

### 3.1 Study Inclusion 

**Fig. 1.**
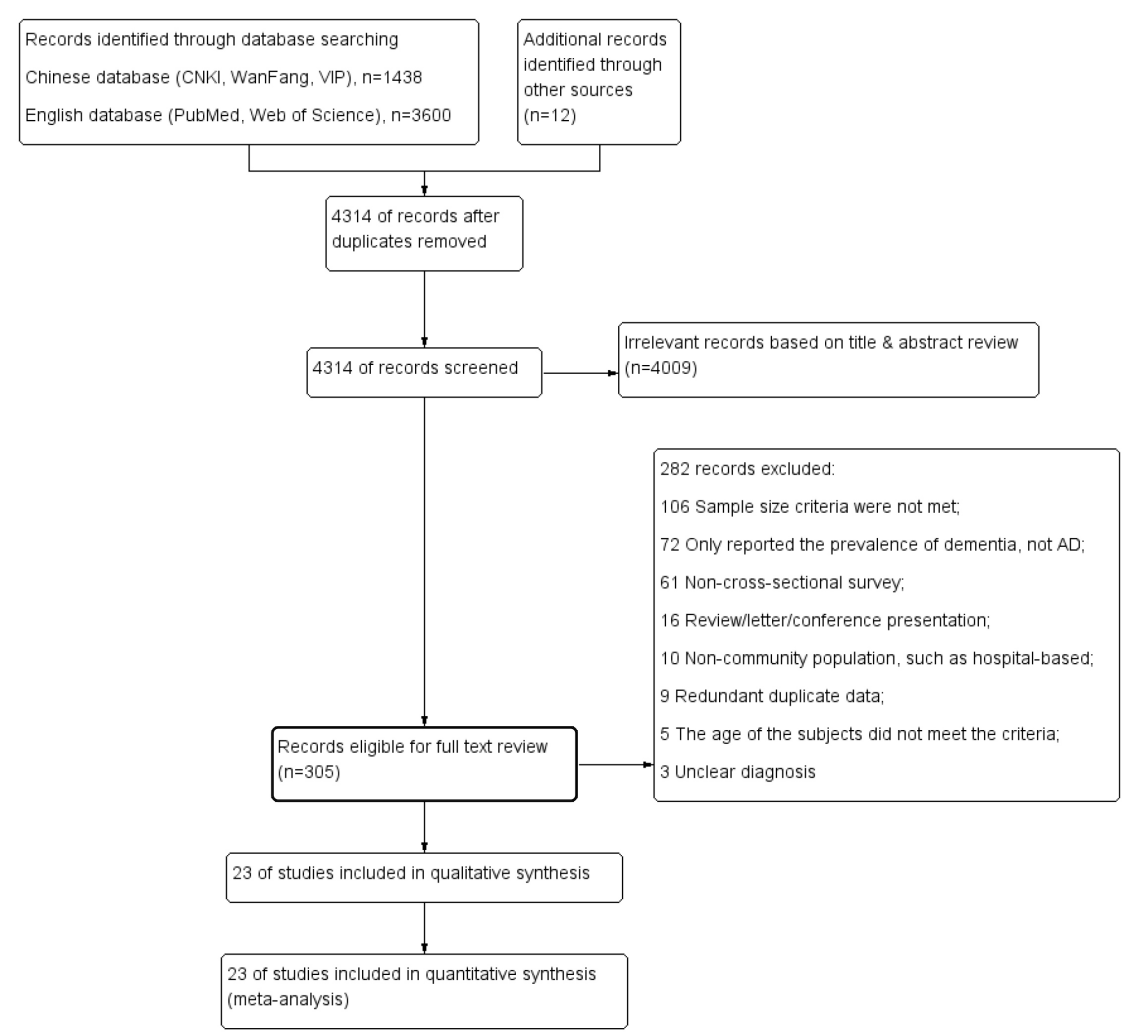
**Flowchart of the literature selection process**. AD, Alzheimer disease.

A total of 5050 studies were initially retrieved. According to the inclusion and exclusion criteria, 23 articles (16 in Chinese and 7 in English) were ultimately included in the detailed analysis. The literature retrieval process is illustrated in Fig. 1. Across these 23 articles, the combined sample size totaled 307,415 individuals, with 13,662 cases of AD being specifically reported. The quality scores of these articles ranged from 4 to 8, and general information of the included articles is presented in Table 1 (Ref. [9, 10, 11, 12, 13, 14, 15, 16, 17, 18, 19, 20, 21, 22, 23, 24, 25, 26, 27, 28, 29, 30, 31]).

**Table 1.** **The characteristics and quality scores of the included studies**.

First author & published year	Region	Diagnostic criteria	Sampling method	Age	No. of subjects	No. of case	AHRQ score
Ding Ding (2014) [10]	Shanghai	NINCDS-ADRDA	census	≥60	3141	113	6
Heming Huang (2014) [11]	Shenzhen	NINCDS-ADRDA	cluster randomized sampling	≥65	3368	177	7
Juan Cheng (2014) [9]	Peking	DSM-IV	multistage stratified random cluster sampling	≥60	3885	59	7
Zhanping Zou (2014) [12]	Zhejiang	MMSE/ICD-10	stratified cluster sampling	≥60	121,949	4795	7
Qiuqin Li (2014) [13]	Zhejiang	MMSE/CCMD-2-R	randomized sampling	≥60	2451	102	4
Jianping Jia (2014) [14]	Changchun, Peking, Zhengzhou, Guiyang, Guangzhou	MMSE	multistage cluster sampling	≥65	10,276	330	6
Chonghui Li (2015) [15]	Tianjin	DSM-IV/NINCDS-ADRDA/NINDS-AIREN	cluster randomized sampling	≥60	2532	144	6
Yong Ji (2015) [16]	Tianjin	NINCDS-ADRDA	no mentioned	≥60	5578	299	6
Jun Liao (2015) [17]	Jiangxi	MMSE/NINCDS-ADRDA	census	≥60	9733	432	4
Li Yang (2016) [18]	Zhejiang	MMSE/NIA-AA	multistage stratified random cluster sampling	≥65	2015	239	6
Gangping Wang (2016) [19]	Gansu	DSM-IV	multistage stratified random cluster sampling	≥60	2416	119	6
Yue Wu (2017) [20]	Jiangsu	NINCDS-ADRDA	stratified cluster random sampling	≥60	4195	205	7
Aiqun Xing (2019) [21]	Hainan	DSM-IV-TR	multistage cluster sampling	≥60	10,000	296	6
Yuzhi Xu (2020) [22]	Peking	MMSE/CCMD-3	convenient sampling	≥60	5901	319	5
Longfei Jia (2020) [23]	12 provinces	NIA-AA	multistage stratified cluster-sampling	≥60	46,011	1801	8
Qiuyan Wang (2021) [24]	Zhejiang	NINCDS-ADRDA	cluster randomized sampling	≥60	2454	109	6
Jianfeng Shao (2021) [25]	Zhejiang	MMSE/ICD-10	stratified cluster random sampling	≥65	5483	301	6
Bin Yuan (2021) [26]	Tibet Autonomous Region	NIA-AA	stratified cluster random sampling	≥60	8000	1046	6
Bin Yuan (2021) [26]	Shandong	NIA-AA	stratified cluster random sampling	≥60	8000	956	6
Xiaoyan Huang (2021) [27]	Zhejiang	DSMIV-TR	multistage cluster sampling	≥60	10,000	431	5
Shige Qi (2021) [28]	Peking, Shanghai, Hubei, Sichuan, Guangxi, Yunnan	MMSE/NINCDS-ADRDA	multistage clustered sampling	≥60	24,117	452	7
Zhetuo Pan (2022) [29]	Zhejiang	NINCDS-ADRDA	multistage sampling	≥60	3152	326	6
Hailing Wang (2022) [30]	Henan	AD8/CSI-D	multistage stratified random cluster sampling	≥65	7326	443	6
Yifei Ren (2024) [31]	Shandong	DSM-IV/NIA-AA	cluster randomized sampling	≥60	5432	168	5

Diagnostic criteria: NINCDS-ADRDA, national institutes of neurological disorders and stroke-Alzheimer’s disease and related disorders association; DSM-IV, the diagnostic and statistical manual of mental disorders, fourth edition; MMSE, the mini-mental state examination; ICD-10, international classification of diseases 9th/10 editions; CCMD-2-R, the Chinese classification of mental disorders; NINDS-AIREN, the national institute of neurological disorders and stroke and association internationale pour la recherche et l’Enseignement en neurosciences; NIA-AA, national institute on aging–Alzheimer’s association guidelines; AD8, Alzheimer disease survey (8 editions); CSI-D, community screening instrument-dementia; AHRQ, agency for healthcare research and quality.

### 3.2 Pooled Prevalence of AD

**Fig. 2.**
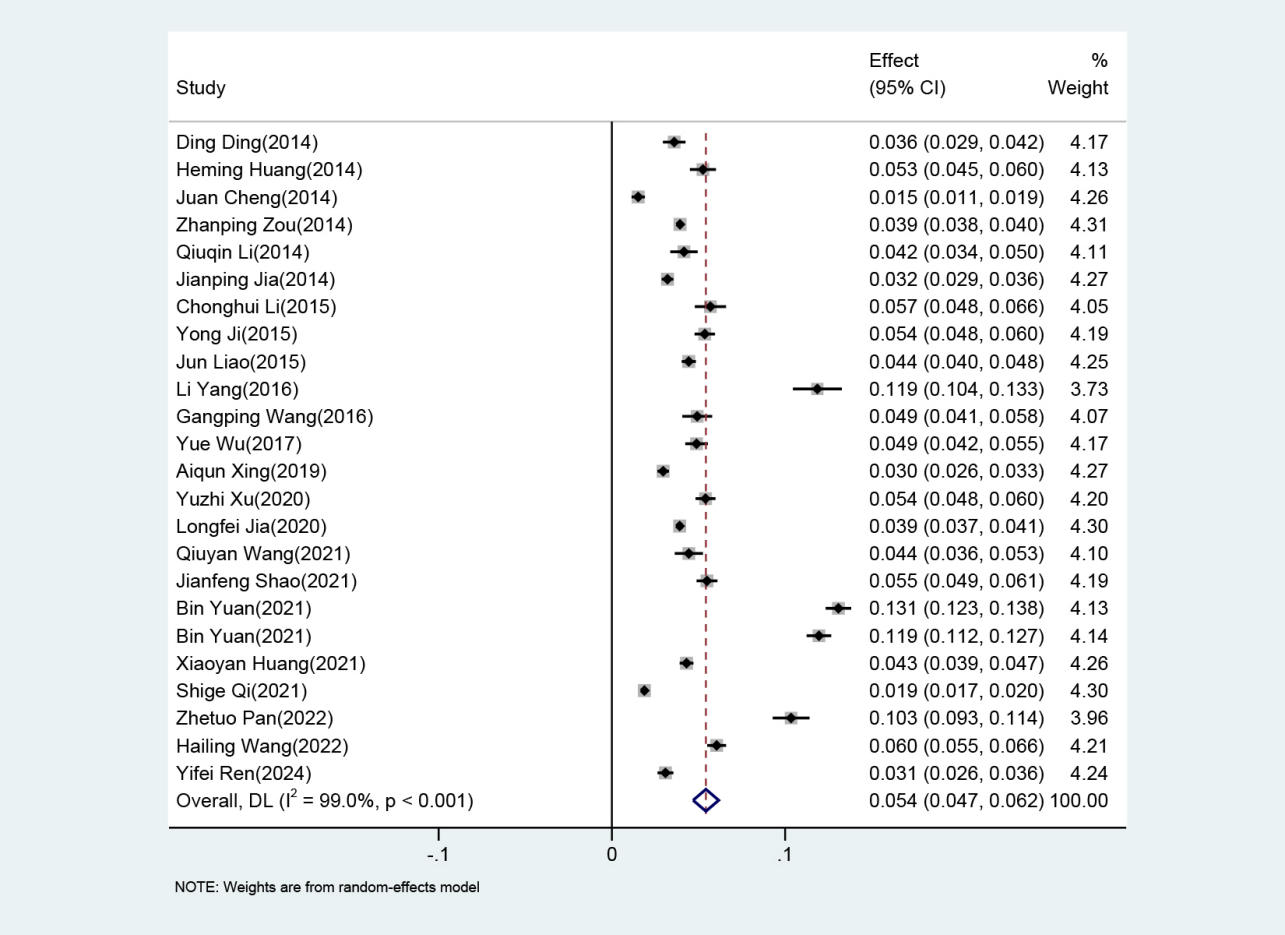
**Forest plot of the prevalence of Alzheimer disease**.

The heterogeneity test of the 23 included studies showed significant heterogeneity (*I*^2^ = 99.0%, *p* < 0.05; Fig. 2). The random-effects model was selected for Meta-analysis, and the results showed that the pooled prevalence of AD was 5.4% (95% CI: 4.7%–6.2%).

**Fig. 3.**
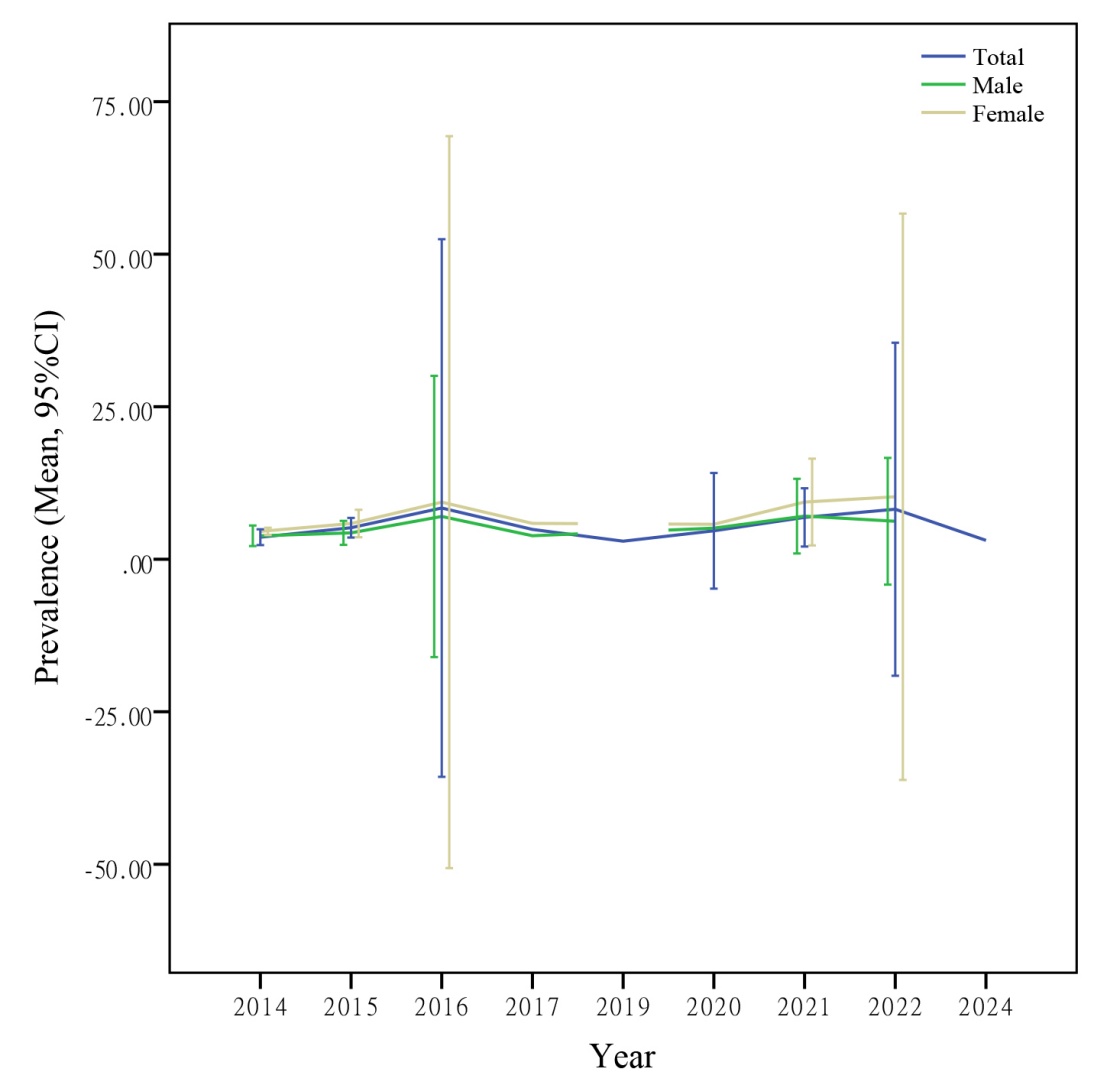
**The prevalence of AD among the elderly in China over the past decade**.

By analyzing the development trend of AD prevalence among older adults in China over the past 10 years, it was found that the prevalence showed a fluctuating trend, among which the prevalence in 2016 and 2022 were relatively high (Fig. 3).

### 3.3 Subgroup Analysis and Meta-regression

Among the 23 included studies, 16 studies explicitly provided the prevalence data for the different genders. The prevalence of AD among females were 7.2% (95% CI: 6.0%–8.4%), significantly higher than the prevalence among males (5.4%, 95% CI: 4.3%–6.4%) (β = 0.016, *p* < 0.001).

The prevalence of AD showed a clear upward trend with increasing age. The prevalence in the 60–69 age group is 3.2% (95% CI: 2.2%–4.2%), 6.2% (95% CI: 4.6%–7.9%) in the 70–79 age group, and as high as 14.3% (95% CI: 11.3%–17.3%) in the ≥80 age group. The regression coefficient β is 0.054 with a *p*-value less than 0.001, suggesting that age is an important factor influencing the prevalence.

In terms of education, the lower the educational level, the higher the prevalence. The prevalence of AD among older adults with primary school education or below was 8.8% (95% CI: 6.6%–11.1%), which was significantly higher than that among those with junior high school education (3.9%, 95% CI: 2.7%–5.2%) and senior high school education or above (3.0%, 95% CI: 2.1%–3.9%).

The prevalence of AD was 5.5% (95% CI: 4.4%–6.6%) for the urban population and 6.6% (95% CI: 4.6%–8.0%) for the rural population. The regression coefficient β is 0.007 with a *p*-value of 0.043, indicating that the regional factor has a certain influence on the prevalence, but it may be weaker compared to other factors. There are differences in prevalence among different geographical regions (Table 2). The prevalence of AD was the highest in the northwestern region (9.0%, 95% CI: 1.0%–17.0%) and lowest in Southern China (5.3%, 95% CI: 4.6%–6.0%). The meta-regression results indicated that geographical region has a significant impact on the prevalence.

**Table 2.** **The subgroup and meta-regression analysis of prevalence of AD among the Chinese elderly population from 2014 to 2024**.

Analysis item		Studies, n	Sample, n	Cases, n	Heterogeneity test		Pooled prevalence (95% CI)	Model	β value	*p* value
					*I*^2^ value (%)	*p* value				
Gender										
	Male	16	102,903	4273	98.3	<0.001	0.054 (0.043–0.064)	Random	0.016	<0.001
	Female	16	116,454	6342	98.3	<0.001	0.072 (0.060–0.084)			
Age (y)										
	60∼69	14	48,319	1644	98.6	<0.001	0.032 (0.022–0.042)	Random	0.054	<0.001
	70∼79	13	37,386	2099	98.3	<0.001	0.062 (0.046–0.079)			
	≥80	13	14,440	1772	97.0	<0.001	0.143 (0.113–0.173)			
Education										
	Primary and below	13	59,500	4123	99.2	<0.001	0.088 (0.066–0.111)	Random	–0.028	<0.001
	Junior high school	10	16,940	557	95.6	<0.001	0.039 (0.027–0.052)			
	High school and above	9	10,730	279	89.7	<0.001	0.030 (0.021–0.039)			
Area										
	Urban	12	182,756	7887	99.0	<0.001	0.055 (0.044–0.066)	Random	0.007	0.043
	Rural	8	39,208	2111	98.9	<0.001	0.066 (0.046–0.080)			
Geographic region										
	Southern	12	177,941	7526	96.8	<0.001	0.053 (0.046–0.060)	Random	0.015	<0.001
	Northern	7	25,896	1777	99.2	<0.001	0.056 (0.032–0.079)			
	Northwestern	2	10,416	1165	99.5	<0.001	0.090 (0.010–0.170)			
Diagnostic criteria										
	NINCDS-ADRDA	9	58,270.00	2257.00	98.6	<0.001	0.051 (0.036–0.065)	Random	0.003	<0.001
	MMSE	8	181,925.00	6970.00	98.9	<0.001	0.049 (0.039–0.059)			
	DSM-IV	6	34,265.00	1217.00	96.7	<0.001	0.037 (0.026–0.048)			
	NIA-AA	5	69,458.00	4210.00	99.6	<0.001	0.088 (0.049–0.126)			
	ICD-10	2	127,432.00	5096.00	96.0	<0.001	0.047 (0.032–0.062)			
	Others^*^	5	25,536.00	1451.00	78.2	<0.001	0.055 (0.049–0.061)			

* Indicates grouping of other single diagnostic criteria into one category.

The prevalence varies under different diagnostic criteria. For example, it is 5.1% (95% CI: 3.6%–6.5%) under the national institutes of neurological disorders and stroke-Alzheimer’s disease and related disorders association (NINCDS-ADRDA) criteria and 4.9% (95% CI: 3.9%–5.9%) under the mini-mental state examination (MMSE) criteria. The regression coefficient β is 0.003 with a *p*-value less than 0.001, suggesting that diagnostic criteria are also a factor influencing the estimation of prevalence.

### 3.4 Sensitivity Analysis and Publication Bias 

Sensitivity analysis was performed using a random-effects model. There were no significant changes in the pooled prevalence when any of the studies were removed (**Supplementary Fig. 1**).

Funnel plots and Egger’s tests were used to assess the publication bias. The funnel plot was asymmetric, indicating a potential publication bias in the combined results (**Supplementary Fig. 2**). Subsequent analysis using Egger’s test confirmed the existence of publication bias in the pooled prevalence of AD (*t *= 2.79, *p *= 0.011) (**Supplementary Figs. 3**,**4**).

## 4. Discussion

Dementia is a syndrome characterized by a progressive decline in cognitive and functional abilities, predominantly affecting individuals aged 60 and above. Based on the underlying pathology, dementia is clinically categorized into the following forms [32, 33]. AD is the leading cause and prototypical form, presenting insidiously and causing progressive memory impairment and cognitive dysfunction with increasing severity over years. Vascular dementia (VaD) is widely recognized as the second most common form of dementia and often co-occurs with other progressive cognitive disorders. Lewy body dementia encompasses Parkinson’s disease dementia and dementia with Lewy bodies, which have similar neuropathological profiles and spectra of clinical symptoms, and are differentiated primarily by the order of motor and cognitive symptom onset. Frontotemporal dementia (FTD) is a rare form of dementia that occurs earlier than other forms, progresses rapidly, and often has a genetic component. As a result of population aging, dementia has emerged as a pressing public health concern. Previous research has predominantly concentrated on the prevalence of dementia among elderly Chinese individuals, whereas the present study focused on elucidating the prevalence of AD specifically.

The results of this study showed that the prevalence of AD among elderly Chinese individuals over the past decade was 5.4% (95% CI: 4.7%–6.2%), which is higher than the findings of another meta-analysis conducted in China in 2020 (3.20%, 95% CI: 3.17%–3.23 %) [34]. It is note-worthy that studies conducted in the United States [35] (5.7%) and Korea [36] (5.7%) have shown a higher prevalence of AD than that in China, while studies from Europe [37] (5.05%) and Japan [38] (3.8%) have indicated a lower prevalence. The global inconsistencies may be attributed to disparities in the genetic, environmental, lifestyle, and diagnostic criteria across individual study [39].

Consistent with previous findings, the prevalence of AD was significantly higher among females [38, 40]. This is related to differences between men and women in terms of physiological, psychological, and social factors [41]. Studies found that sex hormones and sex chromosomes interact with various disease mechanisms during aging, encompassing inflammation, metabolism, and autophagy, leading to unique characteristics in AD progression between men and women [41, 42]. A marked decrease in circulating estrogen levels during menopause may increase the risk of developing AD [43, 44]. In addition, higher levels of mental stress, excessive social roles and caregiving responsibilities, coupled with relatively fewer opportunities for education and career advancement, could contribute to a higher incidence of AD among women [42, 43, 45, 46]. Moreover, because women generally have a longer average lifespan than men, they face a higher risk of developing the disease in their later years.

Multiple epidemiological studies have shown that the incidence of AD significantly increases with age [9, 36, 40]. Aging is a complex and irreversible process that occurs in multiple organs and cell systems. It is manifested by a reduction in the brain’s volume and weight, a loss of synapses, enlargement of the ventricles in specific areas, accompanied by the deposition of senile plaques and the formation of neurofibrillary tangles. These structural and pathological changes in the brain create conditions for the development of AD [39].

This study found a negative correlation between the prevalence of AD and educational level, which is consistent with most previous results [47, 48]. The cognitive reserve model has been proposed to explain the association between higher education level and the lower incidence of AD. Cognitive reserve refers to the brain’s capacity to maintain functional resilience in the presence of AD neuropathology [48]. A shorter duration of education among residents may lead to a lack of sufficient cognitive reserves, subsequently reducing the capacity of the brain to resist damage.

Our results showed that the prevalence of AD was significantly higher in rural areas than in urban areas. There may be some adverse environmental and lifestyle factors in rural areas, including lack of education, unhealthy eating habits, and exposure to pesticides, all of which can negatively affect cognitive function [49]. Furthermore, unbalanced allocation of medical resources affects the prevalence of AD in rural areas. The regional subgroup analyses suggested a large variation in the prevalence of AD in China, which can be attributed to enormous differences in the natural and social environments, economic development, and traditions among different regions. In addition, this study found that variations in diagnostic criteria could affect the pooled effect size, which could be attributed to differences in the definition and judgement methods of AD among different diagnostic criteria.

This meta-analysis was conducted to reveal the prevalence of AD in the elderly population in China over the past decade. The results showed that AD has become a major public health problem in China. Therefore, the implementation of effective preventive measures is imperative. At the individual level, the elderly should strengthen comprehensive prevention strategies by enhancing cognitive training through activities such as reading, learning new skills, and playing board games to stimulate brain function and improve cognitive abilities. Maintaining healthy weight management through balanced diets, caloric control, and increased dietary fiber consumption is equally essential. Regular engagement in aerobic exercise, including walking and jogging, can promote cerebral blood circulation and metabolic function. Cultivating a positive mindset via social interactions and hobbies helps alleviate negative emotions, while ensuring a nutrient-rich diet with adequate proteins, vitamins, and brain-beneficial components—such as omega-3 fatty acids from fish and antioxidants from fruits and vegetables—provides foundational support for cognitive health.

At the societal level, the government and relevant departments should increase healthcare investment and optimize resource allocation to strengthen primary healthcare services, particularly ensuring equitable access to quality medical care for elderly populations in rural and remote areas. Concurrently, enhancing public education to raise awareness of Alzheimer’s disease and establishing robust social support systems are critical to providing assistance to affected families and fostering a dementia-inclusive society.

This study has several limitations. First, there was considerable heterogeneity in the combined results. The subgroup analysis and meta-regression indicated that gender, age, education level, urban/rural, Geographic region, and diagnostic criteria significantly moderated the effect size. However, substantial residual heterogeneity remained, suggesting unmeasured factors (e.g., have other disease, family history, living or marital status) that might contribute to the heterogeneity. Second, all the studies included in this research were cross-sectional studies, and there were relatively few high-quality studies. After removing studies with low AHRQ-score, heterogeneity remained to *I*^2^ = 99.1%. However, sensitivity analysis revealed that there were no significant changes in the pooled prevalence when the low AHRQ-score studies were removed (**Supplementary Table 1**). As mentioned in many other meta-analyses, conducting meta-analyses on cross-sectional studies will inevitably face the problem of relatively large heterogeneity [50, 51]. Third, there was a publication bias in this study. After the trim-and-fill method analysis, it was found that the pooled effect size decreased to 3.6% (95% CI: 2.8%–4.4%) (**Supplementary Table 2**). Therefore, it is impossible to accurately capture the changing trends of AD solely through cross-sectional studies. We believe that the results of this study should be interpreted with caution. Moreover, we hold the view that it is highly necessary to conduct longitudinal studies in the future to comprehensively understand the development trends of AD among the elderly in China.

## 5. Conclusion

With the accelerating aging population in China, the risk of Alzheimer’s disease (AD) among older adults is projected to escalate significantly, necessitating proactive responses from both individuals and governments to address this pressing issue through timely interventions.

## Availability of Data and Materials

The data are obtained from major medical journal databases. The full dataset and data analysis code are available from the corresponding author.

## Supplementary Material

Supplementary material associated with this article can be found, in the online version, at https://doi.org/10.31083/RN36394.

## References

[1] Wortmann M (2012). Dementia: a global health priority - highlights from an ADI and World Health Organization report. *Alzheimer’s Research & Therapy*.

[2] First MB, Reed GM, Hyman SE, Saxena S (2015). The development of the ICD-11 Clinical Descriptions and Diagnostic Guidelines for Mental and Behavioural Disorders. *World Psychiatry: Official Journal of the World Psychiatric Association (WPA)*.

[3] Rujing R, Peng Y, Jinlei Q (2021). China Alzheimer’s disease report 2021. *Journal of Diagnostics Concepts & Practice*.

[4] Wang YQ, Jia RX, Liang JH, Li J, Qian S, Li JY (2019). Dementia in China (2015-2050) estimated using the 1% population sampling survey in 2015. *Geriatrics & gerontology international*.

[5] Page MJ, Moher D, Bossuyt PM, Boutron I, Hoffmann TC, Mulrow CD (2021). PRISMA 2020 explanation and elaboration: updated guidance and exemplars for reporting systematic reviews. *BMJ (Clinical Research Ed.)*.

[6] Stang A (2010). Critical evaluation of the Newcastle-Ottawa scale for the assessment of the quality of nonrandomized studies in meta-analyses. *European Journal of Epidemiology*.

[7] Dong Y, Yan Y, Zhou J, Zhou Q, Wei H (2023). Evidence on risk factors for knee osteoarthritis in middle-older aged: a systematic review and meta-analysis. *Journal of Orthopaedic Surgery and Research*.

[8] Higgins JPT, Thompson SG, Deeks JJ, Altman DG (2003). Measuring inconsistency in meta-analyses. *BMJ (Clinical Research Ed.)*.

[9] Cheng J, Xu N, Ma Y, Wei X, Li X (2014). Epidemiological investigation of Alzheimer’s disease and cognitive impairment and analysis of related risk factors in Beijing. *Journal of Ningxia Medical University*.

[10] Ding D, Zhao Q, Guo Q, Meng H, Wang B, Yu P (2014). The Shanghai Aging Study: study design, baseline characteristics, and prevalence of dementia. *Neuroepidemiology*.

[11] Huang H, Chen S, Zhao Y, Zou L, Zhang R (2014). Analysis of the prevalence and influencing factors of Alzheimer’s disease among the elderly in Shenzhen community. *Chinese Journal of Practical Nervous Diseases*.

[12] Zou Z, Chen L, Zhou L, Shen J, Zhang F (2014). Survey on prevalence of Alzheimer’s disease in Haining city. *Chinese Journal of General Practice*.

[13] Li Q (2014). Analyze and Study the Regional Infection Situation of Alzheimer’s Disease. *Journal of Zhejiang Chinese Medical University*.

[14] Jia J, Wang F, Wei C, Zhou A, Jia X, Li F (2014). The prevalence of dementia in urban and rural areas of China. Alzheimer’s & Dementia: the Journal of the Alzheimer’s Association. *Alzheimer’s & Dementia: the Journal of the Alzheimer’s Association*.

[15] Li C, Yang L, Zhao L, Su Z, Pan P, Wang Y (2015). Epidemiological investigation on the prevalence of senile dementia among the patients served by a community hospital in Beichen District, Tianjin City. *Practical Preventive Medicine*.

[16] Ji Y, Shi Z, Zhang Y, Liu S, Liu S, Yue W (2015). Prevalence of dementia and main subtypes in rural northern China. *Dementia and Geriatric Cognitive Disorders*.

[17] Liao J, Huang H, Yan J, Ma J, Tao X, Liao X (2015). The prevalence of Alzheimer’s disease in Nanchang community and its influencing factors. *Chinese Journal of Gerontology*.

[18] Yang L, Jin X, Yan J, Jin Y, Yu W, Wu H (2016). Prevalence of dementia, cognitive status and associated risk factors among elderly of Zhejiang province, China in 2014. *Age and Ageing*.

[19] Wang G, Pei G, Jie R, Ding Z, Zhang Y (2016). Prevalence of dementia in the people aged 60 and older of Tianshui. *Journal of International Psychiatry*.

[20] Wu Y, Cheng Z, Bao Z, Fan J, Tang L, Guo T (2017). Survey on the status of Alzheimer’s disease and psychological health of family caregivers in Wuxi. *Chinese Preventive Medicine*.

[21] Xing A, Ji X, Zhou S (2019). Analysis of prevalence and related factors of senile dementia in Sanya area. *Chinese Journal of Integrative Medicine on Cardio-Cerebrovascular Disease*.

[22] Xu Y, Liu X, Yin Z, Deng Y (2020). Prevalence of Alzheimer’s disease in Fengtai District, Beijing. *South China Journal of Preventive Medicine*.

[23] Jia L, Du Y, Chu L, Zhang Z, Li F, Lyu D (2020). Prevalence, risk factors, and management of dementia and mild cognitive impairment in adults aged 60 years or older in China: a cross-sectional study. *The Lancet. Public Health*.

[24] Wang Q, Zhu Y (2021). The prevalence of senile dementia in Quzhou city and its risk factors. *China Modern Doctor*.

[25] Shao J, Zhang Y, Shi J (2021). Prevalence of Alzheimer’s Disease and Its Risk Factors in Fuyang District of Hangzhou. *Zhejiang Medical Education*.

[26] Yuan B, Madunzhu M, Danzhuoga C, Zhao Y, Huo Q, Bu M (2021). Incidence of AD in Tibet plateau and inland plain areas. *Chinese Journal of Geriatric Heart Brain and Vessel Diseases*.

[27] Huang X, Zhu Y (2021). Researches on the prevalence, risk factors and economic burden of senile dementia in Quzhou city. *China Modern Doctor*.

[28] Qi S, Yin P, Zhang H, Zhang Q, Xiao Y, Deng Y (2021). Prevalence of Dementia in China in 2015: A Nationwide Community-Based Study. *Frontiers in Public Health*.

[29] Pan Z, Zheng X, Shao X, Mao Y (2022). Epidemiological characteristics of Alzheimer’s disease in people aged 60-85 years in Zhejiang Province. *Practical Preventive Medicine*.

[30] Wang H, Yao F, Guo Z, Wang Y, Dong J, Wang Z (2022). Current situation and influencing factors of Alzheimer’s disease in the elderly aged 65 and above in Henan Province. *Modern Preventive Medicine*.

[31] Ren Y, Li Y, Tian N, Liu R, Dong Y, Hou T (2024). Multimorbidity, cognitive phenotypes, and Alzheimer’s disease plasma biomarkers in older adults: A population-based study. Alzheimer’s & Dementia: the Journal of the Alzheimer’s Association. *Alzheimer’s & Dementia: the Journal of the Alzheimer’s Association*.

[32] Wilbur J (2023). Dementia: Dementia Types. *FP Essentials*.

[33] Raz L, Knoefel J, Bhaskar K (2016). The neuropathology and cerebrovascular mechanisms of dementia. *Journal of Cerebral Blood Flow and Metabolism: Official Journal of the International Society of Cerebral Blood Flow and Metabolism*.

[34] Cui L, Hou NN, Wu HM, Zuo X, Lian YZ, Zhang CN (2020). Prevalence of Alzheimer’s Disease and Parkinson’s Disease in China: An Updated Systematical Analysis. *Frontiers in Aging Neuroscience*.

[35] Steenland K, Goldstein FC, Levey A, Wharton W (2016). A Meta-Analysis of Alzheimer’s Disease Incidence and Prevalence Comparing African-Americans and Caucasians. *Journal of Alzheimer’s Disease: JAD*.

[36] Kim KW, Park JH, Kim MH, Kim MD, Kim BJ, Kim SK (2011). A nationwide survey on the prevalence of dementia and mild cognitive impairment in South Korea. *Journal of Alzheimer’s Disease: JAD*.

[37] Niu H, Álvarez-Álvarez I, Guillén-Grima F, Aguinaga-Ontoso I (2017). Prevalence and incidence of Alzheimer’s disease in Europe: A meta-analysis. *Neurologia (Barcelona, Spain)*.

[38] Sekita A, Ninomiya T, Tanizaki Y, Doi Y, Hata J, Yonemoto K (2010). Trends in prevalence of Alzheimer’s disease and vascular dementia in a Japanese community: the Hisayama Study. *Acta Psychiatrica Scandinavica*.

[39] Breijyeh Z, Karaman R (2020). Comprehensive Review on Alzheimer’s Disease: Causes and Treatment. *Molecules (Basel, Switzerland)*.

[40] Matthews FE, Arthur A, Barnes LE, Bond J, Jagger C, Robinson L (2013). A two-decade comparison of prevalence of dementia in individuals aged 65 years and older from three geographical areas of England: results of the Cognitive Function and Ageing Study I and II. *Lancet (London, England)*.

[41] Aggarwal NT, Mielke MM (2023). Sex Differences in Alzheimer’s Disease. *Neurologic Clinics*.

[42] Lopez-Lee C, Torres ERS, Carling G, Gan L (2024). Mechanisms of sex differences in Alzheimer’s disease. *Neuron*.

[43] Torromino G, Maggi A, De Leonibus E (2021). Estrogen-dependent hippocampal wiring as a risk factor for age-related dementia in women. *Progress in Neurobiology*.

[44] Yeung CHC, Au Yeung SL, Kwok MK, Zhao JV, Schooling CM (2023). The influence of growth and sex hormones on risk of alzheimer’s disease: a mendelian randomization study. *European Journal of Epidemiology*.

[45] Palpatzis E, Akinci M, Garcia-Prat M, Blennow K, Zetterberg H, Quijano-Rubio C (2025). Grief and Economic Stressors by Sex, Gender, and Education: Associations With Alzheimer Disease-Related Outcomes. *Neurology*.

[46] Johansson L (2014). Can stress increase Alzheimer’s disease risk in women?. *Expert Review of Neurotherapeutics*.

[47] Seyedsalehi A, Warrier V, Bethlehem RAI, Perry BI, Burgess S, Murray GK (2023). Educational attainment, structural brain reserve and Alzheimer’s disease: a Mendelian randomization analysis. *Brain: a Journal of Neurology*.

[48] Rosselli M, Uribe IV, Ahne E, Shihadeh L (2022). Culture, Ethnicity, and Level of Education in Alzheimer’s Disease. *Neurotherapeutics: the Journal of the American Society for Experimental NeuroTherapeutics*.

[49] Liu X, Wang G, Cao Y (2024). The prevalence of mild cognitive impairment and dementia among rural dwellers: A systematic review and meta-analysis. *Geriatric Nursing (New York, N.Y.)*.

[50] Song P, Zhang Y, Yu J, Zha M, Zhu Y, Rahimi K (2019). Global Prevalence of Hypertension in Children: A Systematic Review and Meta-analysis. *JAMA Pediatrics*.

[51] Valesan LF, Da-Cas CD, Réus JC, Denardin ACS, Garanhani RR, Bonotto D (2021). Prevalence of temporomandibular joint disorders: a systematic review and meta-analysis. *Clinical Oral Investigations*.

