# Supplementary material for: Trends in the Prevalence and Development of Alzheimer’s Disease Among the Elderly Chinese Population: A Systematic Review
Source: Rev Neurol. 2025 Jul 28;80(6):36394. doi: 10.31083/RN36394 (PMC12326449; doi:10.31083/RN36394)
Supplement: Supplementary file 1 [file 1576-6578-80-6-36394-s1.zip › supplement tables.pdf]

Supplementary table 1 sensitive analysis (deletion of low-quality studies)

Analysis item	Studies	Heterogeneity test		Pooled prevalence (95% CI)
		I^2 value (%)	*P* value	
AHRQ≥4	24	99.0	＜0.001	0.054(0.047-0.062)
AHRQ≥5	22	99.1	＜0.001	0.055(0.047-0.063)
AHRQ≥6	19	99.2	＜0.001	0.057(0.049-0.066)

Supplement table 2 Filled meta-analysis

	No. of studies	Method	Pooled prevalence (95% CI)	Heterogeneity test	
				Q	*P* value
Meta-analysis					
	24	Fixed	0.038(0.037-0.039)	2274.48	＜0.001
		Random	0.054(0.047-0.062)		
Filled Meta-analysis					
	33	Fixed	0.035(0.034-0.035)	4096.47	＜0.001
		Random	0.036(0.028-0.044)		
