# Supplementary figures and images for: Trends in the Prevalence and Development of Alzheimer’s Disease Among the Elderly Chinese Population: A Systematic Review
Source: Rev Neurol. 2025 Jul 28;80(6):36394. doi: 10.31083/RN36394 (PMC12326449; doi:10.31083/RN36394)

# Meta-analysis estimates, given named study is omitted

| Lower CI Limit

○ Estimate

| Upper CI Limit

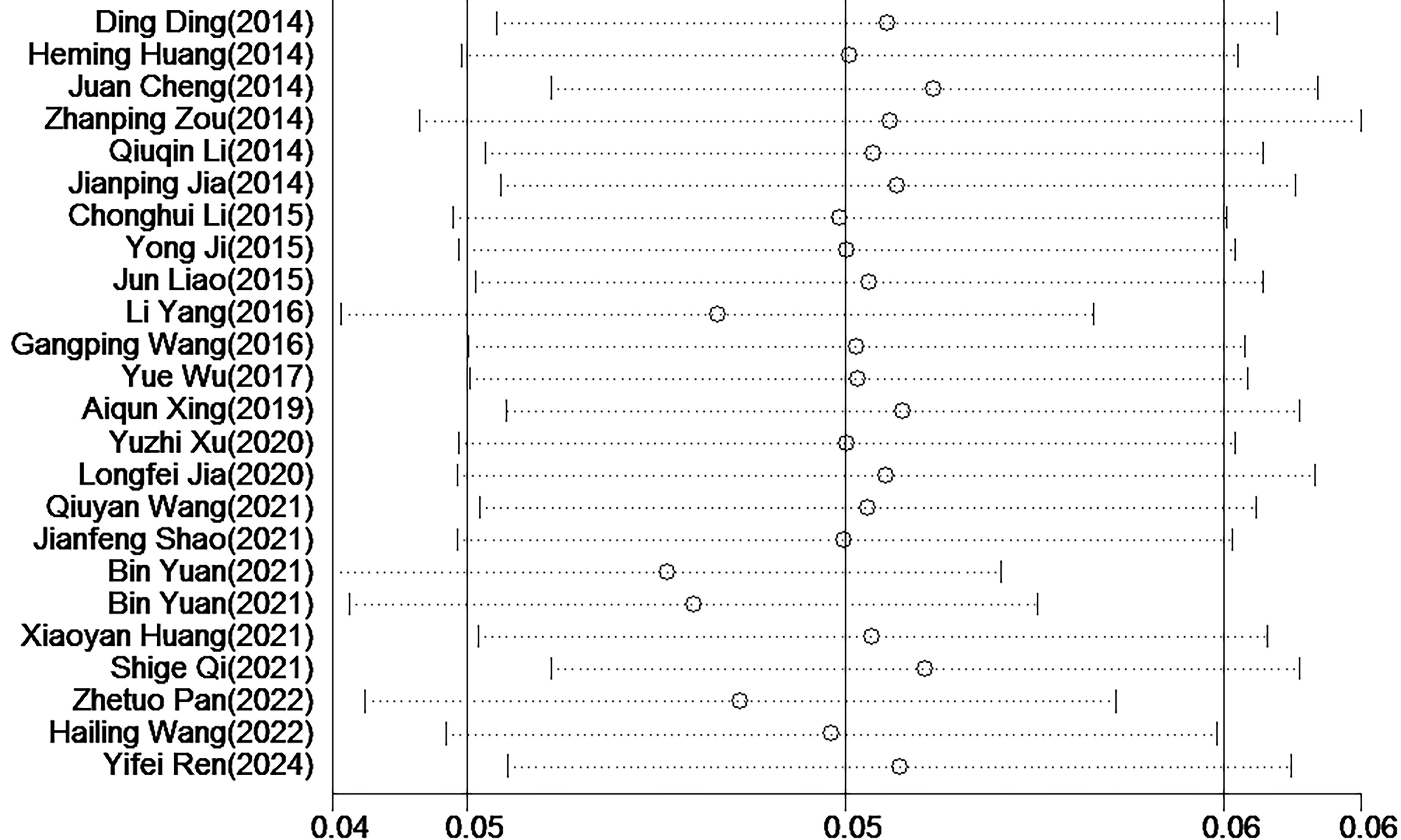

Supplement: Supplementary file 1 [file 1576-6578-80-6-36394-s1.zip › Supplementary figure 1 sensitive analysis.pdf]

Funnel plot with pseudo 95% confidence limits

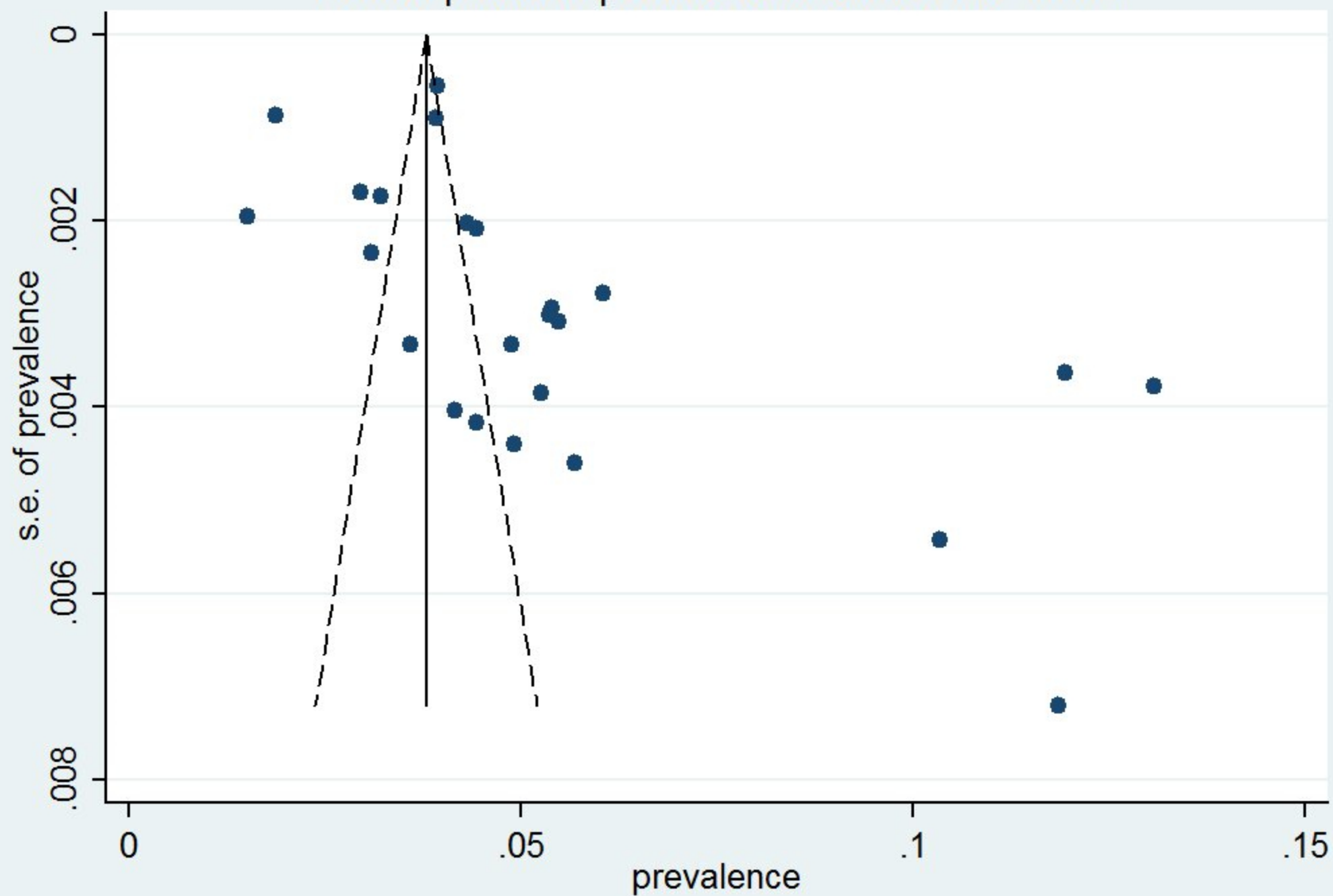

Supplement: Supplementary file 1 [file 1576-6578-80-6-36394-s1.zip › Supplementary figure 2 Funnel plot.pdf]

Egger's publication bias plot

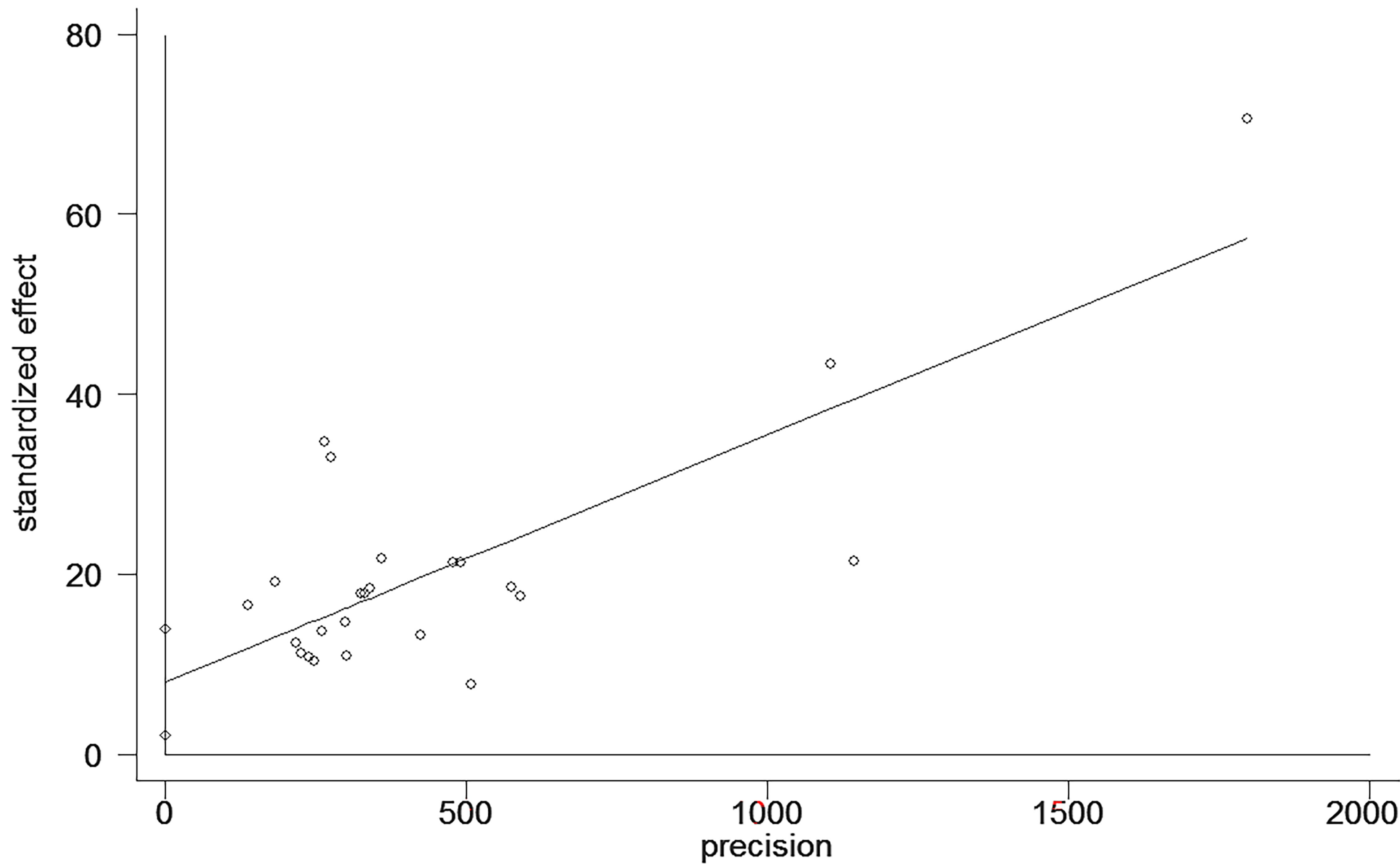

Supplement: Supplementary file 1 [file 1576-6578-80-6-36394-s1.zip › Supplementary figure 3 Egger's publication bias plot.pdf]

Filled funnel plot with pseudo 95% confidence limits

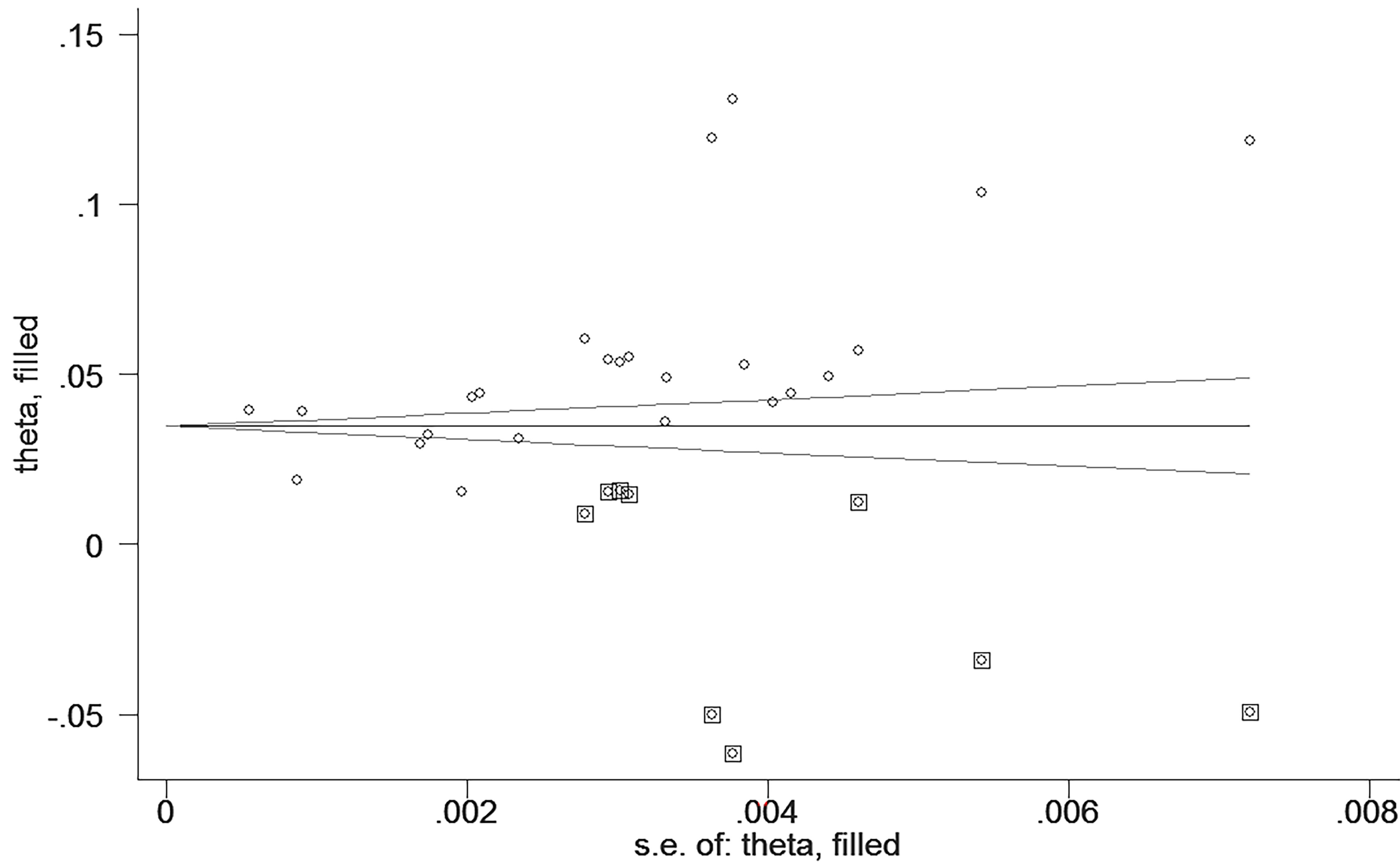

Supplement: Supplementary file 1 [file 1576-6578-80-6-36394-s1.zip › Supplementary figure 4 Filled funnel plot.pdf]
